# Occult Perforated Gangrenous Gallbladder Found on Magnetic Resonance Cholangiopancreatography

**DOI:** 10.7759/cureus.15754

**Published:** 2021-06-19

**Authors:** Kevin Parza, Pooja Patel, Nicolina Scibelli, Jilian R Sansbury

**Affiliations:** 1 Internal Medicine, Grand Strand Medical Center, Myrtle Beach, USA

**Keywords:** perforated gallbladder, gangrenous cholecystitis, cholecystectomy, necrotic gallbladder, occult perforated gallbladder, cholecystitis, liver ultrasound, magnetic resonance cholangiopancreatography (mrcp), computed tomography, atypical abdominal pain

## Abstract

Acute gangrenous cholecystitis is a life-threatening disease that is most often diagnosed intraoperatively and can be missed on mildly symptomatic patients without the proper imaging modality. We present a case of a 69-year-old male with a history of hypertension, hyperlipidemia, and type 2 diabetes, and a recent right pontine infarct that arrived with 3 out of 10 right-sided abdominal pain. His liver ultrasound and computed tomography (CT) with contrast demonstrated acute cholecystitis. He was initially worked up conservatively and was scheduled for an elective cholecystectomy per surgery recommendation. However erring on the side of caution, the medical team had ordered a magnetic resonance cholangiopancreatography (MRCP), which demonstrated perforated gangrenous cholecystitis. Of note, the imaging modalities were ordered within a 24-hour window. The patient’s antibiotics were promptly broadened, and he was emergently sent to the operating room. Moving forward, we will identify atypical clinical presentations of gangrenous cholecystitis and consider ordering an MRCP when clinical suspicion remains high and initial imaging is inconclusive. Perforated gangrenous cholecystitis is a severe disease and can cause rapid demise if not identified and treated early.

## Introduction

Gangrenous cholecystitis (GC) arises from acute cholecystitis (AC) when there is necrosis and possible perforation of the gallbladder wall. A gallstone prevents blood flow inducing ischemia and conversion to necrotic tissue [1]. Of all the AC cases that enter the hospital, about 30% of them can convert to GC [2].

A recent study had shown the risk of death for GC is between 15% and 50% if left untreated [3]. Current guidelines recommend observing for disease progression in patients with AC. This includes monitoring vital signs such as fever, or changes in pain severity on a daily basis. However, given the high mortality of GC and risk of more invasive surgery, the medical community is constrained by the lack of a widely accepted scoring system or radiographic findings indicative of GC. GC is typically diagnosed intra-operatively or through histopathologic samples, which involves a significant time delay in treatment for asymptomatic or minimally symptomatic patients [4].

Ultrasound remains the first-line modality and it can have findings of thickened gallbladder wall which can be nonspecific for an inflamed gallbladder such as seen in AC [5]. A CT abdomen with contrast can be a second-line alternative but may also have non-specific findings including intraluminal gases [5]. Due to the fact that MRCP has a higher sensitivity for detecting GC, it can be quite useful when the findings from the other two modalities are inconclusive or can even be used as a first-line in the detection of gallbladder pathologies [6].

## Case presentation

The patient is a 69-year-old male with a past medical history of hypertension, hyperlipidemia, type 2 diabetes, and a recent cerebrovascular accident (CVA) (right pontine infarct) who arrived from an outside rehabilitation center as a stroke alert with a chief complaint of left-sided weakness and loss of consciousness, and right-sided abdominal pain. The patient reported being in physical therapy when his left-sided weakness worsened causing him to have a mechanical fall and land on his right side. After the fall, the patient was found to have altered mental status as per bystanders prompting the admission. Upon presentation, the patient was alert and oriented and complained of left-sided weakness and right quadrant pain that was 3 out of 10 in severity. The patient stated his right quadrant pain started four weeks ago following discharge from a prior stroke workup. The patient denied any inciting factors to his pain, other than exertion.

**Figure 1:**
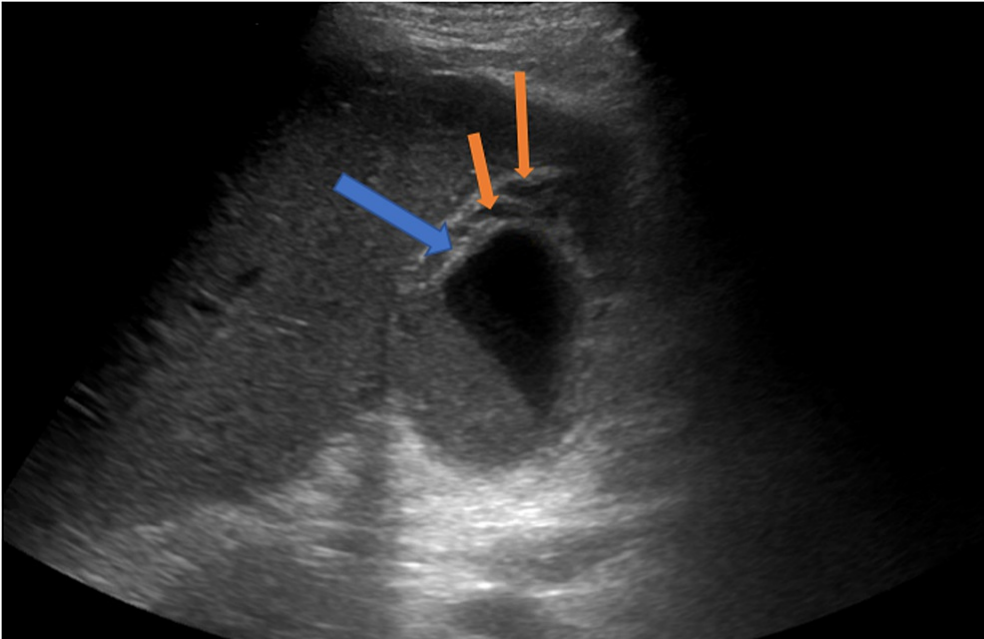
Ultrasound (trans gallbladder view) of the gallbladder consistent with acute cholecystitis. Thickened gallbladder wall (blue arrow) and pericholecystic fluid (orange arrows).

**Figure 2:**
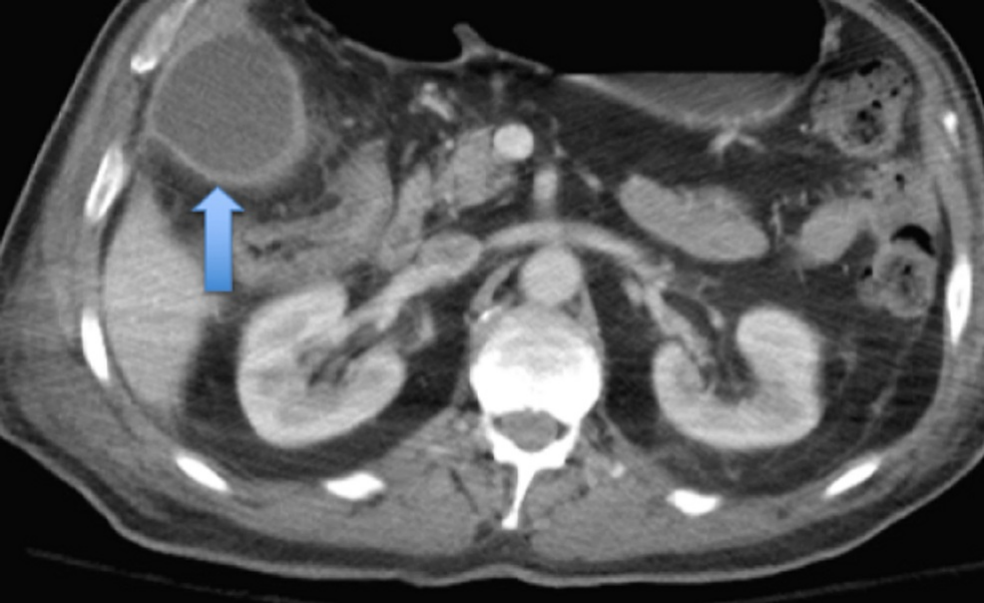
Computed tomography (CT) abdomen and pelvis with contrast (axial view). Dilated and inflamed gallbladder consistent with acute cholecystitis. Gallbladder wall thickening (blue arrow).

**Figure 3:**
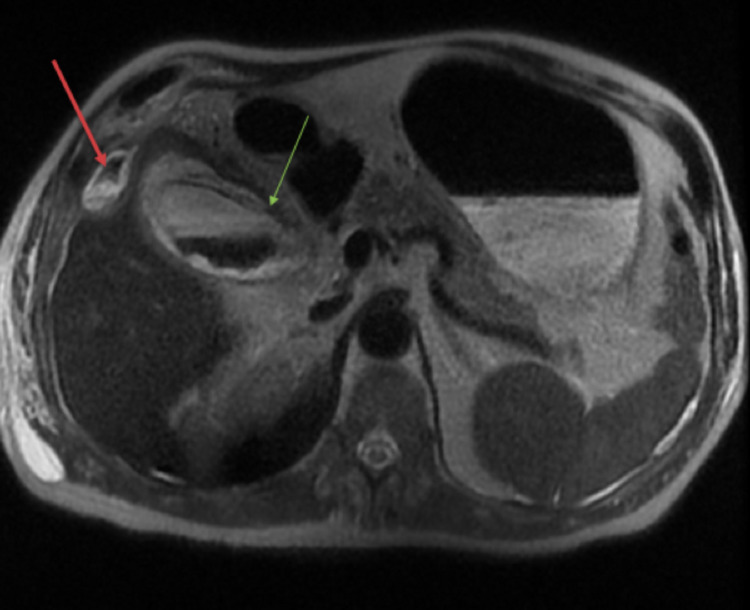
Magnetic resonance (MR) T2-weighted (axial view) findings consistent with perforated gangrenous cholecystitis. Note there is fundal wall discontinuity (green arrow) and subjacent right upper quadrant and subcapsular abscesses/phlegmonous change (red arrow).

On admission, he was hemodynamically stable and did not meet systemic inflammatory response (SIRS) criteria. Physical exam was pertinent for mild tenderness on his right upper quadrant. Pertinent labs included a white blood cell count of 11.8 thousand/mL, alkaline phosphatase 259 U/L, aspartate aminotransferase 259 U/L, lipase 721 U/L, and C-reactive protein 18 mg/dL. His hepatitis panel was negative. The patient underwent a syncope and stroke workup which was found to be unremarkable. An abdominal ultrasound showed AC with pericholecystic fluid (Figure 1). His CT abdomen and pelvis with contrast was ordered per radiologist recommendation and it showed AC with fluid communication with the liver, but no abscess (Figure 2). The hepatobiliary surgery team was consulted, and they had recommended conservative management and elective cholecystectomy. On day 2, the patient complained of 4 out of 10 abdominal pain and was unable to tolerate any food. His mild leukocytosis resolved. A magnetic resonance cholangiopancreatography (MRCP) was ordered to investigate for biliary tree pathology given the elevated lipase. The MRCP showed perforated gangrenous cholecystitis with subcapsular abscess (Figure 3).

**Figure 4:**
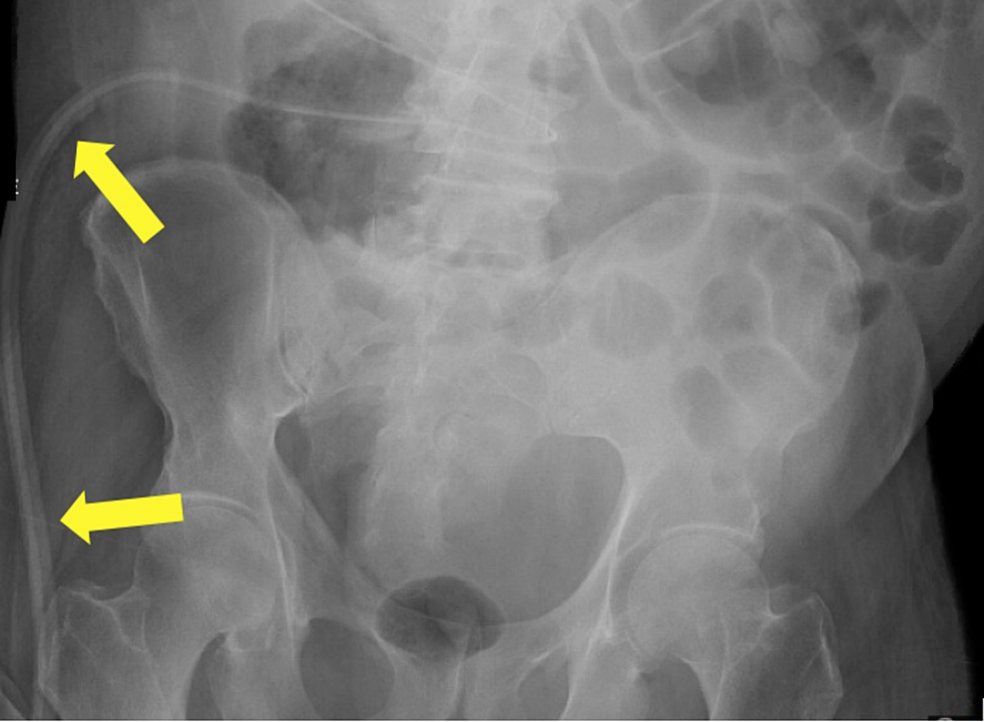
Abdominal x-ray (AP supine view) demonstrates a Jackson Pratt (JP) drain after open cholecystectomy. Note the drain (yellow arrows). AP - anteroposterior

The surgical team was re-consulted, and the patient underwent an emergent laparoscopic cholecystectomy, where intraoperative and histopathology reports both confirmed GC. During his surgery, the patient was reported to have non-identifiable surgical planes with an indistinct cystic duct and artery indicating advanced disease. He then had a Jackson Pratt (JP) drain in place (Figure 4), which was later removed during his two-week follow-up at the outpatient clinic. The patient had no postoperative complications.

## Discussion

GC is the most common complication of AC. Fever, right upper quadrant pain, and jaundice consistent with Charcot’s triad and a positive Murphy’s sign are the most common presentation of a complicated AC [7]. However, atypical GC can present without the above findings and instead have minimal right upper quadrant tenderness or even a complete absence of pain [8].

Of all the risk factors for GC, the most prevalent was the presence of diabetes [9]. Patients with a history of diabetes can often have polyneuropathy, which may symptomatically mask lethal pathologies such as myocardial ischemia and ischemic colitis [10,11]. This phenomenon is suggested by the inability of motor and sensory nerves to transmit signals in the setting of diabetic polyneuropathy. Patients are noted to have a decrease in growth factors which aid in the rebuilding of damaged nerves [12].

When ruling out complications of AC, it is useful to risk stratify the patient in order to prioritize the need for emergent surgery. Charcot’s triad has been a well-used screening tool to aid surgeons with pre-operative planning. However, in a recent study, Charcot’s Triad had a sensitivity of only 36.3%, which deemed it of having the limited clinical utility [13].

While imaging modalities are most useful in diagnosing AC, there has not yet been a gold standard for diagnosing GC. According to a study published in March 2007, both ultrasound and CT have higher specificity, but low sensitivity for identifying acute GC [5]. Due to its low cost and bedside convenience, ultrasound has become a popular first-line for detecting pathologies of the biliary tract [14]. In terms of detecting AC, ultrasounds have a 90%-95% sensitivity and 78%-80% specificity [15]. GC on ultrasound typically has a wall thickness of at least 5.1 mm and or pericholecystic fluid [7], which are nonspecific for GC and can also be found on AC [16].

Patients who present to the hospital with generalized abdominal pain most often undergo a CT exam due to its efficiency and cost. However, the imaging modality is generally accepted as a second-line diagnostic radiographic tool to differentiate gallbladder pathologies [17]. CT has a sensitivity of 91.7% and specificity of 99.1% for AC. However, CT only has a sensitivity of 29.3% and 96% specificity for GC. Findings most specific to GC on a CT abdomen are intraluminal gases and irregular walls [18].

It has been suspected that 15%-30% of patients with acute biliary disorders require MR imaging, including MRCP, which allows for a comprehensive and detailed evaluation of the biliary system. According to Watanabe et al., MRCP has a major advantage over ultrasonography (US) and CT in that it is able to delineate magnitudes of inflammation, determine the existence or lack of necrosis and or abscess, and also other complications of the biliary tract [5]. Unlike the CT scan, MRCP does not require reliance on deterioration by abdominal gas to demarcate structures [17]. Instead through the implementation of T2-weighted imaging and T1-fat suppression, the MRCP is able to delineate wall irregularity more efficiently and generate cross-sectional images [17]. Massimo et al. recommend that urgent MRCP should be one of the initial imaging modalities obtained in the emergency room upon patient presentation. Not only can MRCP detect findings seen in uncomplicated cystitis such as gallbladder distention, impacted stones in the cystic duct; it can also detect complications of AC such as GC, gallbladder perforation, and abscesses [19].

## Conclusions

GC most typically presents with right upper quadrant pain. However, it can present atypically with minimal pain or even asymptomatically. Due to the low sensitivity for GC, imaging modalities should not be limited to US and CT abdomen for the diagnosis of GC. Therefore, it is important for clinicians to have a high index of suspicion for GC by utilizing risk stratification tools and MRCP when prior imaging modalities are inconclusive. Doing so will ensure an opportunity for the timely intervention of a disease that has the potential to have fatal outcomes if left untreated.
